# Urgent Conservation Actions Are Needed for Qinling Lenok *Brachymystax lenok tsinlingensis* Li, 1966: Enlightenment From Model Simulations

**DOI:** 10.1002/ece3.71427

**Published:** 2025-05-26

**Authors:** Yuebing Zhou, Xianghong Dong, Tao Ju, Lei Gan, Zhenlu Wang, Yuxi Lian, Peng Zhang, Xiongfeng Bai, Qing Liu, Shuhai Zhang, Jiyuan Liu, Tao Xiang, Lei Shi, Haibo Jiang, Jian Shao, Miao An

**Affiliations:** ^1^ Department of Fisheries Sciences, College of Animal Science Guizhou University Guiyang China; ^2^ Guangxi Academy of Marine Sciences Guangxi Academy of Sciences Nanning China; ^3^ College of Life Science Anqing Normal University Anqing China; ^4^ State Key Laboratory of Water Resources Engineering and Management Wuhan University Wuhan China; ^5^ College of Animal Science Shanxi Agricultural University Jinzhong China; ^6^ Nanjing Institute of Geography and Limnology Chinese Academy of Sciences Nanjing China; ^7^ Institute for Ecological Research and Pollution Control of Plateau Lakes, School of Ecology and Environmental Science Yunnan University Kunming China

**Keywords:** global warming, human pressure, MaxEnt algorithm, mountain cold‐water fishes, species distribution models (SDMs)

## Abstract

The Qinling lenok *Brachymystax lenok tsinlingensis* Li, 1966, an endemic to China and South Korea, is a rare protected species. Its unique requirements to habitat have made this fish extraordinarily fragile when faced with human pressures and global warming. Hence, predicting and understanding the potential influence of human pressures and global warming on this fish's spatial distribution is quite critical for the conservation and management of the species. To do so, based on its occurrence records and current as well as future environmental dataset, this study constructed a Maximum Entropy (MaxEnt) model for the species to analyze how its potential suitable areas (PSAs) would respond to global warming and human pressures (3 Global Climate Models ×2 Shared Socioeconomic Pathways ×3 future time nodes). The results showed that: (1) the MaxEnt model had strong generalization or transferability ability (AUC > 0.90), was highly reliable to predicting the current and future PSAs of the species; (2) Mean Temperature of Driest Quarter (BIO9), Human Population Density (Pop), Elevation (Elev), and Mean Temperature of Wettest Quarter (BIO8) were the salient environmental factors (given in descending order by significance); (3) the present PSAs for this fish were mainly distributed in Europe, Asia, and North America, and with the intensification of global changes, these areas in all continents would shrink on a large scale, and their distribution centroids would move towards northwest. Based on the above, a series of proposals for conservation and management of the fish were put forward so as to alleviate the loss of this relict species' habitats in the future.

## Abstract 2

How Chinese salmonids would respond to stressors has been first assessed; Qinling lenok would be in peril; Urgent conservation actions are needed for this rare and endangered fish.

## 1 Introduction

As an important component of the aquatic ecosystem, rivers are one of the habitats which hold the most abundant fish diversity on our blue planet. Although covering less than 1% of earth's land surface, rivers possess 31.08% of all known fish species of the world (Barbarossa et al. 2021; Fricke et al. 2023). Moreover, it is foreseeable that, with the advancement of related research, more novel riverine fish species will be discovered in the future (Zanata et al. 2024). Although plentiful river fish play a vital role in maintaining ecosystem balance, human health, food security, fishermen's livelihoods, and cultures around the world (Holmlund and Hammer 1999; WWF et al. 2021; Youn et al. 2014), they are being faced with more and more severe threats due to growing anthropogenic activities and global warming (Barbarossa et al. 2021; Lee et al. 2023). For instance, a groundbreaking study titled *Human Impacts on Global Freshwater Fish Biodiversity* (Su et al. 2021) highlighted a striking reality: over half of the world's rivers—spanning 40% of Earth's continental area and 37% of total river length—have experienced a drastic decline in fish species diversity; by contrast, only those in less than 14% of the world's continental surface or the world's river length were close to unchanged (Su et al. 2021). In such a context, how to protect and manage the sprites dwelling in flowing rivers, especially those that are more sensitive to global change (even subtle environmental changes can trigger strong responses of these species) as a result of living at high altitudes (Urban 2024), has drawn great attention from many organizations and groups, including conservation biologists.

The Qinling lenok *Brachymystax lenok tsinlingensis* Li, 1966 is a typical one of these. The fish, a member of Salmoniformes, Salmonidae, mainly feeds on invertebrates such as aquatic insects (Zhang et al. 2022), and is now distributed in the alpine streams of the Qinling Mountains of China and the Nakdong River of South Korea (Li 1984; Osinov 2024; Yu and Kwak 2015). A more interesting thing is that this fish is a relic species after *Brachymystax* migrated from north to south during the quaternary glacial period, a representative of the landlocked piedmont cold‐water fishes (Li 1984), as well as one of the geographically southernmost salmonids (Xia et al. 2017). However, because of human activities over the past half century, such as overfishing, water pollution, and habitat degradation, its wild population went through a dramatic decline (Hong et al. 2024; Ko et al. 2011; Qin 2014; Wang 2008). Therefore, it was listed as protected wildlife in both China and South Korea (Jang et al. 2017; Yue and Chen 1998). What is more worrisome is that the aforementioned climate change‐induced rises of water temperature and changes of seasonal patterns, etc. by many human stressors have put this species in even greater danger (P. Li 2022; Peng et al. 2021; Xia et al. 2021). Thus, to predict and understand the influence of human pressures and global warming on this fish's spatial distribution pattern is quite a critical scientific question for the conservation and management of the species. But, published works on this fish only focused on its basic biology (Gao 2014; Xue et al. 2013), ecology (Ko et al. 2021; Sun 2014), physiology (Fang et al. 2023; Xiong et al. 2019), ethology (Deng et al. 2024), molecular biology (Guo et al. 2023; Si et al. 2012; Wen et al. 2020), phylogenetic systematics and adaptive evolution (Osinov 2024; Zhu et al. 2022), and phylogeography or zoogeography (Liu et al. 2015; Li 2022). Even though understanding how a species' spatial distribution pattern will respond to numerous pressure sources is very important to its conservation and management (Carpenter et al. 1993), to date, such research on the *B. lenok tsinlingensis* is still missing.

Species distribution models (SDMs), a powerful ecological and statistical machine learning method, that can analyze and predict species' potential suitable areas (PSAs) under different environmental conditions and extract the main environmental drivers affecting its spatial distribution, is a common approach to solve the above issue (Newbold 2018; Shipley et al. 2022). Such tools involve a large number of machine learning algorithms, which estimate the mathematical relationship between species occurrence data and relevant environmental variables by fitting them according to a specific algorithm, and project this functional relationship into different time and space so as to obtain the species' PSAs in other landscapes or time nodes of interest, including past, present, and future periods (Guisan and Zimmermann 2000; Zhu et al. 2013). In general, these predictions reflect species' preference to habitat in the form of probability, and are usually interpreted as the species' occurring probability in a certain place, habitat suitability, or species abundance (Elith et al. 2006; Li et al. 2013). Among all these alternative algorithms, Maximum Entropy (MaxEnt; Phillips et al. 2006) is undoubtedly one of the most popular methods for modeling species distribution. This mainly attributes to its unique advantages: (1) it has a supporting implementation software, which simplifies the modeling process by only “click” rather than sophisticated programming skills; (2) it has rich features (linear, quadratic, product, hinge, threshold, and classification) to be capable of building a range of models from simple to complex (Low et al. 2021) to meet different needs; (3) robust operation on small samples (Pearson et al. 2007). Therefore, this algorithm has been widely applied (Elith et al. 2011) and performs well on many occasions (Pearson et al. 2007; Phillips et al. 2006), especially in applications involving small samples, such as the research on the conservation of rare and endangered species (Bai et al. 2018).

With the above in view, this study aimed to: (1) develop a MaxEnt model for *B. lenok tsinlingensis*; (2) identify the leading environmental variables pertaining to its differential spatial distribution; (3) predict the effects of global environment change on the spatial distribution pattern of its PSAs. The results are of important ecological significance and practical value, since they can not only provide a scientific basis for the conservation of this species, but also give conducive references to other rare and endangered fish, for example, *Hucho bleekeri* Kimura, 1934.

## 2 Materials and Methods

### 2.1 Species Occurrence Data

**FIGURE 1:**
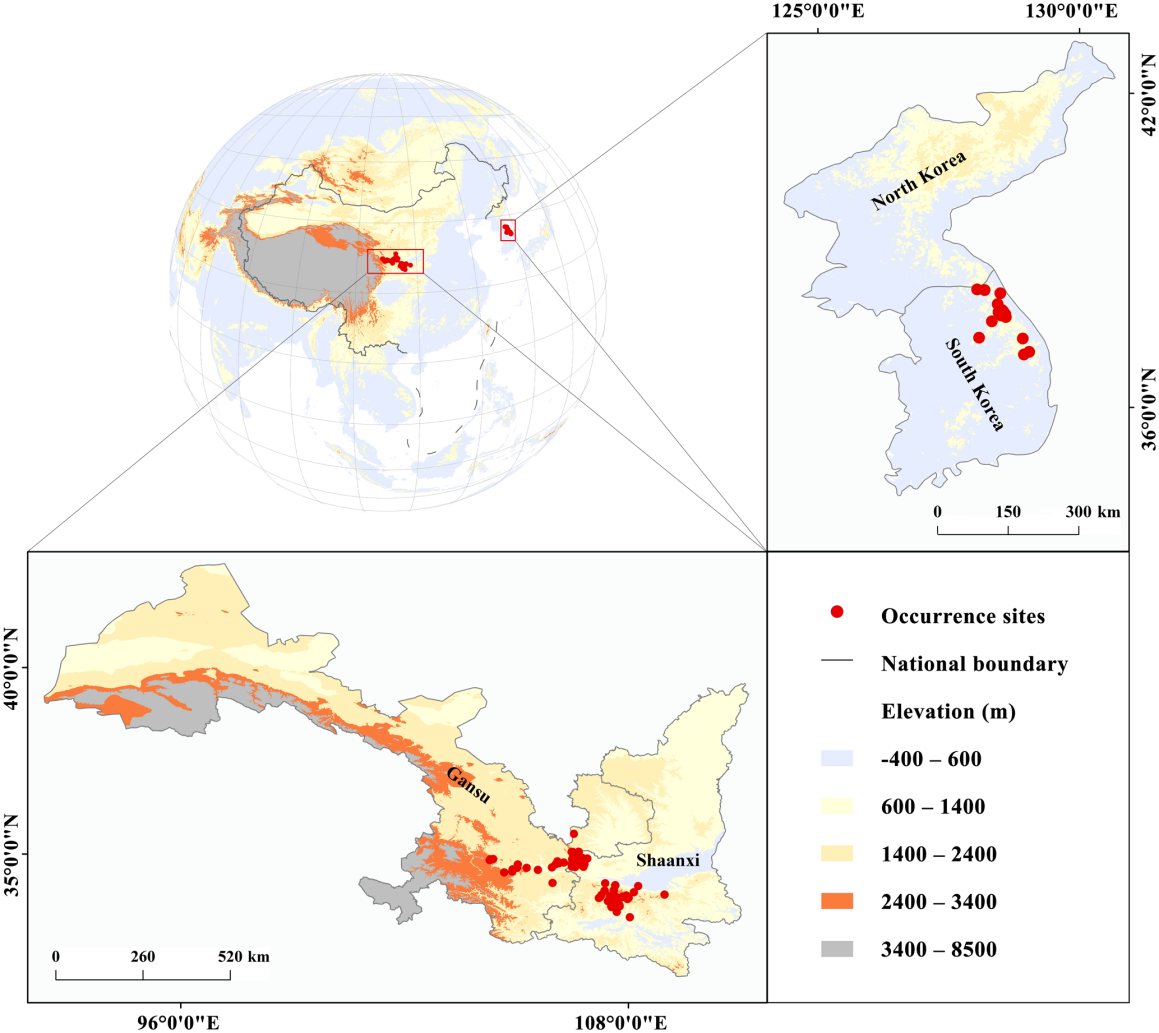
Map illustrating spatial distribution points of Qinling lenok *Brachymystax lenok tsinlingensis* Li, 1966.

Species occurrence points of *B. lenok tsinlingensis* were gathered systematically based on three data sources as follows: (1) China National Knowledge Infrastructure (https://www.cnki.net); (2) Google Scholar (https://scholar.google.com); (3) Web of Science (https://www.webofscience.com/wos). The data types contained journal articles, monographies, and dissertations, etc., and the data languages contained Chinese, English, and Korean. After excluding those with unclear sampling locations, incomplete information records, nonstandard matching of Latin names, the duplications, and the obvious false entries, a total of 180 valid species occurrence points were collected in this study. It is noteworthy that at this time, these data still had sampling bias or spatial auto‐correlation caused by the spatial clustering effect, which would probably reduce the effectiveness of subsequent species distribution modeling (Steen et al. 2021). Hence, before further processing, we used a so‐called spatial thinning method to lessen the sampling bias hidden in the species presence data or to weaken their spatial auto‐correlation (Boria et al. 2014; Riul et al. 2013), that is, to ensure that at most one record was contained in each environmental grid cell (ca. 21 km^2^ at the equator) with 2.5 arc‐minutes spatial resolution (when more than one record dropped in one environmental grid cell, the computer would randomly delete the redundant points; Steen et al. 2021). Finally, 125 presence records were retained for subsequent MaxEnt modeling. These presence records were heterogeneously distributed in parts of China and South Korea (Figure 1).

### 2.2 Environmental Variables

Based on the existing knowledge of *B. lenok tsinlingensis* (Deng et al. 2024; Sun 2014; Tao et al. 2024; Wu et al. 2017; Yoon et al. 2015) and data availability, we preliminarily selected Human Population Density (Pop; 1), Elevation (Elev; 1), and bioclimatic variables (WorldClim; 19), a total of 21 factors as candidate explanatory variables for MaxEnt modeling in this study (Table 1). Among them, Pop and bioclimatic variables are dynamic variables (varying with time and scenario), while Elev is a static variable (not varying with time and scenario). The reason why static variables are considered in addition to dynamic variables is that Stanton et al. (2012) found that static variables can effectively enhance the generalization or transferability ability of SDMs. Certainly, this study did not put all the 21 environmental variables in analysis, but filtered them before modeling, as in many previous works, in order to eliminate multi‐collinearity among different environmental variables and obviate MaxEnt's overfitting and overparameterization (De Marco and Nóbrega 2018; Liu et al. 2019). Specifically, we selected environmental variables with a Pearson correlation coefficient less than 0.8 in absolute value and a variance inflation factor not more than 10. As a result, 11 explanatory variables were retained for follow‐up analysis: Elev, Pop, Mean Diurnal Range (BIO2), Isothermality (BIO3), Mean Temperature of Wettest Quarter (BIO8), Mean Temperature of Driest Quarter (BIO9), Precipitation of Wettest Month (BIO13), Precipitation of Driest Month (BIO14), Precipitation Seasonality (BIO15), Precipitation of Warmest Quarter (BIO19), and Precipitation of Coldest Quarter (BIO19).

**TABLE 1:** Detail information about the variables used in the current study.

Datasets	Codes	Brief descriptions	Units	Sources
Elevation	**Elev**	**Elevation**	**m**	https://lta.cr.usgs.gov/GTOPO30
Population	**Pop**	**Human Population Density**	**person**/**km** ^**2**^	https://doi.org/10.7927/q7z9‐9r69
WorldClim	BIO1	Annual Mean Temperature	°C	https://www.worldclim.org/
	**BIO2**	**Mean Diurnal Range** (**Mean of monthly** (**max temp**—**min temp**))	**°C**	https://www.worldclim.org/
	**BIO3**	**Isothermality** (**BIO2**/**BIO7**) (×**100**)	**—**	https://www.worldclim.org/
	BIO4	Temperature Seasonality (standard deviation ×100)	°C	https://www.worldclim.org/
	BIO5	Max Temperature of Warmest Month	°C	https://www.worldclim.org/
	BIO6	Min Temperature of Coldest Month	°C	https://www.worldclim.org/
	BIO7	Temperature Annual Range (BIO5–BIO6)	°C	https://www.worldclim.org/
	**BIO8**	**Mean Temperature of Wettest Quarter**	**°C**	https://www.worldclim.org/
	**BIO9**	**Mean Temperature of Driest Quarter**	**°C**	https://www.worldclim.org/
	BIO10	Mean Temperature of Warmest Quarter	°C	https://www.worldclim.org/
	BIO11	Mean Temperature of Coldest Quarter	°C	https://www.worldclim.org/
	BIO12	Annual Precipitation	mm	https://www.worldclim.org/
	**BIO13**	**Precipitation of Wettest Month**	**mm**	https://www.worldclim.org/
	**BIO14**	**Precipitation of Driest Month**	**mm**	https://www.worldclim.org/
	**BIO15**	**Precipitation Seasonality** (**Coefficient of Variation**)	**—**	https://www.worldclim.org/
	BIO16	Precipitation of Wettest Quarter	mm	https://www.worldclim.org/
	BIO17	Precipitation of Driest Quarter	mm	https://www.worldclim.org/
	**BIO18**	**Precipitation of Warmest Quarter**	**mm**	https://www.worldclim.org/
	**BIO19**	**Precipitation of Coldest Quarter**	**mm**	https://www.worldclim.org/

*Note:* “—” means dimensionless. Variables with bold font were selected or retained for subsequential modeling analysis.

### 2.3 Global Climate Models and Shared Socioeconomic Pathways

In order to reduce the uncertainty caused by individual Global Climate Models (GCMs) and improve the accuracy of our model's prediction, this study selected three GCMs (MRI‐ESM2‐0, MPI‐ESM1‐2‐HR, and EC‐Earth3‐Veg) with good performance in East Asia from Coupled Model Intercomparison Project Phase 6 (CMIP6) to generate bioclimatic variables that would be used for MaxEnt modeling in the three future time nodes (2030s (2020–2040), 2050s (2040–2060) and 2070s (2060–2080)) (Goberville et al. 2015; Lu et al. 2022; Zhou et al. 2021). Although there are multiple Shared Socioeconomic Pathways (SSPs) that can be used to match the foregoing three GCMs, to make the problem simple, we just selected two extreme ones for analysis: the sustainability path (SSP1‐2.6: under this path, the global forest coverage will increase and the dependence on resources and fossil energy will decrease; radiative forcing maintained at 2.6 w/m^2^ by 2100) and the fossil‐fueled development path (SSP5‐8.5: under this path, economic development will become energy‐intensive and greenhouse gas emission will increase; radiative forcing reached 8.5 w/m^2^ by 2100).

### 2.4 MaxEnt Modeling

To initiate species distribution modeling for *B. lenok tsinlingensis*, we called “megaSDM” package (version 1.1.0; Shipley et al. 2022) in R (version 4.2.1; R Core Team 2022). Specific practices were as follows: firstly, this package was used to produce the background points (BPs) required for MaxEnt modeling, that is, 10,000 spatially‐constrained BPs were randomly generated using the “combination method” in this package, and 50% of them were randomly sampled from the entire study area, while the rest were taken randomly from the buffers around each true occurrence point (the buffer radius is twice as many as the 95% quantile of the minimum distance from each occurrence point to all other points) (Phillips and Dudík 2008; Shipley et al. 2022). Then, we executed environmental subsampling to BPs and species occurrence records, respectively, with the aim of obtaining data sets with less bias or spatial auto‐correlation for the final modeling by conducting multiple environmental filtering to the data on the basis of spatial thinning (Castellanos et al. 2019; Kramer‐Schadt et al. 2013; Varela et al. 2014). Secondly, we performed MaxEnt algorithm fitting following occupation standards or common practice, that is, (1) divided randomly the post‐environmental‐filtering occurrence data into training (80%) and testing (20%) sets; (2) selected linear, quadratic, hinge, product, and threshold features depending on the number of species occurrence records (Elith et al. 2011); (3) invoked jackknife and permutation tests to identify the environmental variables that contributed to the MaxEnt model and were sensitive to species distribution; (4) chose the area under the receiver operating characteristic (ROC) curve (AUC; ranges from 0.5 to 1.0; independent from thresholds and prevalence; Raes and ter Steege 2007) to evaluate the model's generalization or transferability ability (generally, that the ROC curve is above the diagonal means the model has good generalization or transferability ability. Otherwise, it means the opposite and a reverse result should be adopted. To summarize, the further the AUC curve is away from the diagonal, the more reliable the result is, otherwise, the less reliable the result is (Fielding and Bell 1997; Raes and ter Steege 2007)); (5) drawn marginal response curves of the species' habitat suitability to all the 11 explanatory variables. Certainly, this modeling workflow was repeated 10 times in order to erase the uncertainties by data‐partitioning and obtain a more robust model. Based on the 10 sub‐models, we finally developed an ensemble model (Shipley et al. 2022) and transformed the continuous results produced by the ensemble model into the simpler binary variables (i.e., 0 or 1) via maximizing the True Skill Statistics (i.e., sensitivity + specificity − 1) of the testing set (Allouche et al. 2006; Liu et al. 2016). More details about the modeling processes can refer to the help document of “megaSDM” and relevant papers (Shipley et al. 2022). All data analysis and graphics in this study were performed in R 4.2.1 (R Core Team 2022) and ArcGIS 10.8.1 (ESRI 2018); if not specified, all statistics were expressed as mean ± standard deviation (SD).

## 3 Results

### 3.1 Model Performance

**FIGURE 2:**
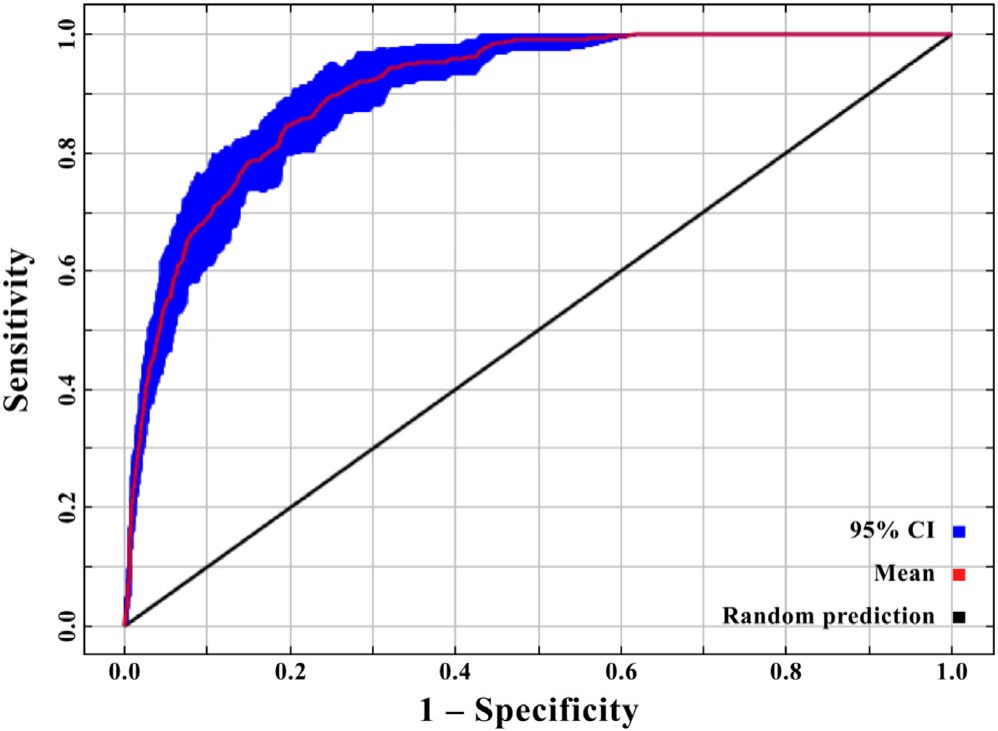
Model performance or generalization ability (*n* = 10, i.e., 10 repetitions) indicating by the area under the receiver operating characteristic curve (AUC). SD is the abbreviation of standard deviation. CI stands for confidence interval.

The results showed that the ROC curve was at the upper left of the diagonal, and the AUC score was 0.91 ± 0.02, which indicated that we created an excellent MaxEnt model (AUC > 0.90) for *B. lenok tsinlingensis*. This meant that our ensemble model held strong generalization or transferability ability and was reliable for predicting the current and future spatial distribution of the species in question (Figure 2).

### 3.2 Variables' Relative Contribution and Sensitivity

**FIGURE 3:**
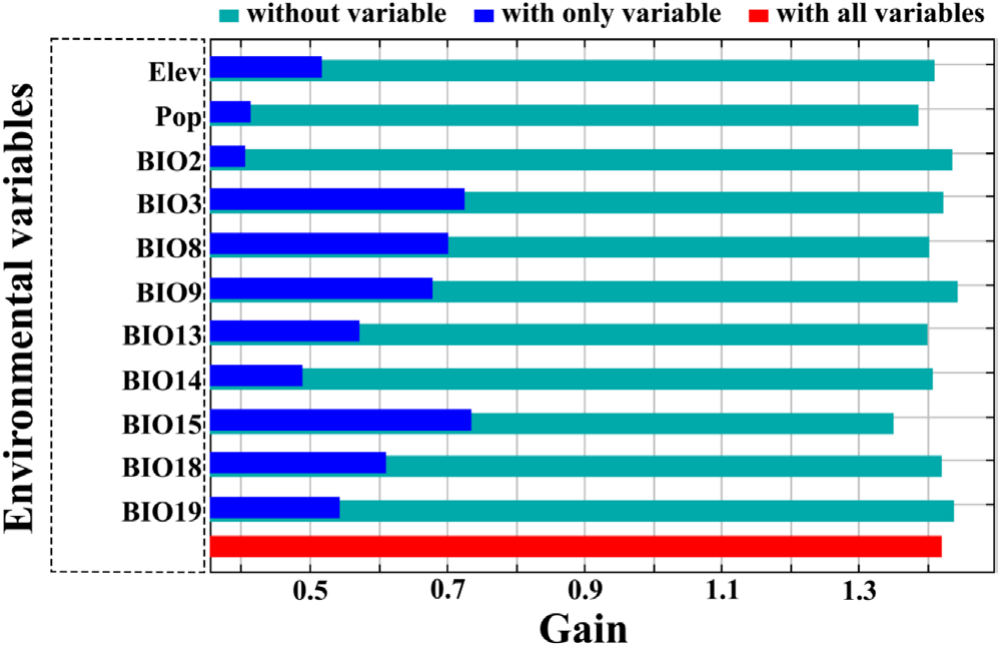
Average variable importance (*n* = 10, i.e., 10 repetitions) relating to the spatial distribution of *B. lenok tsinlingensis* determined by jackknife test. This picture was affiliated with the testing dataset. Abbreviations of these environmental variables are presented in Table 1.

The results revealed that, of the selected 11 explanatory variables, BIO3 (contributing: 25.5%), BIO15 (17.6%), Elev (14.5%), BIO8 (11.9%), BIO9 (8.7%), BIO18 (8.7%), and Pop (7.5%; given in descending order; the same below) were the 7 most influential ones to the spatial distribution of *B. lenok tsinlingensis*, and they cumulatively contributed 94.4% (Figure 3). By contrast, the variables to which the fish was most sensitive in the process of habitat selection were BIO9 (17.6%), Pop (14.4%), Elev (12.6%), and BIO8 (12.0%).

### 3.3 Current and Future PSAs Changes

**FIGURE 4:**
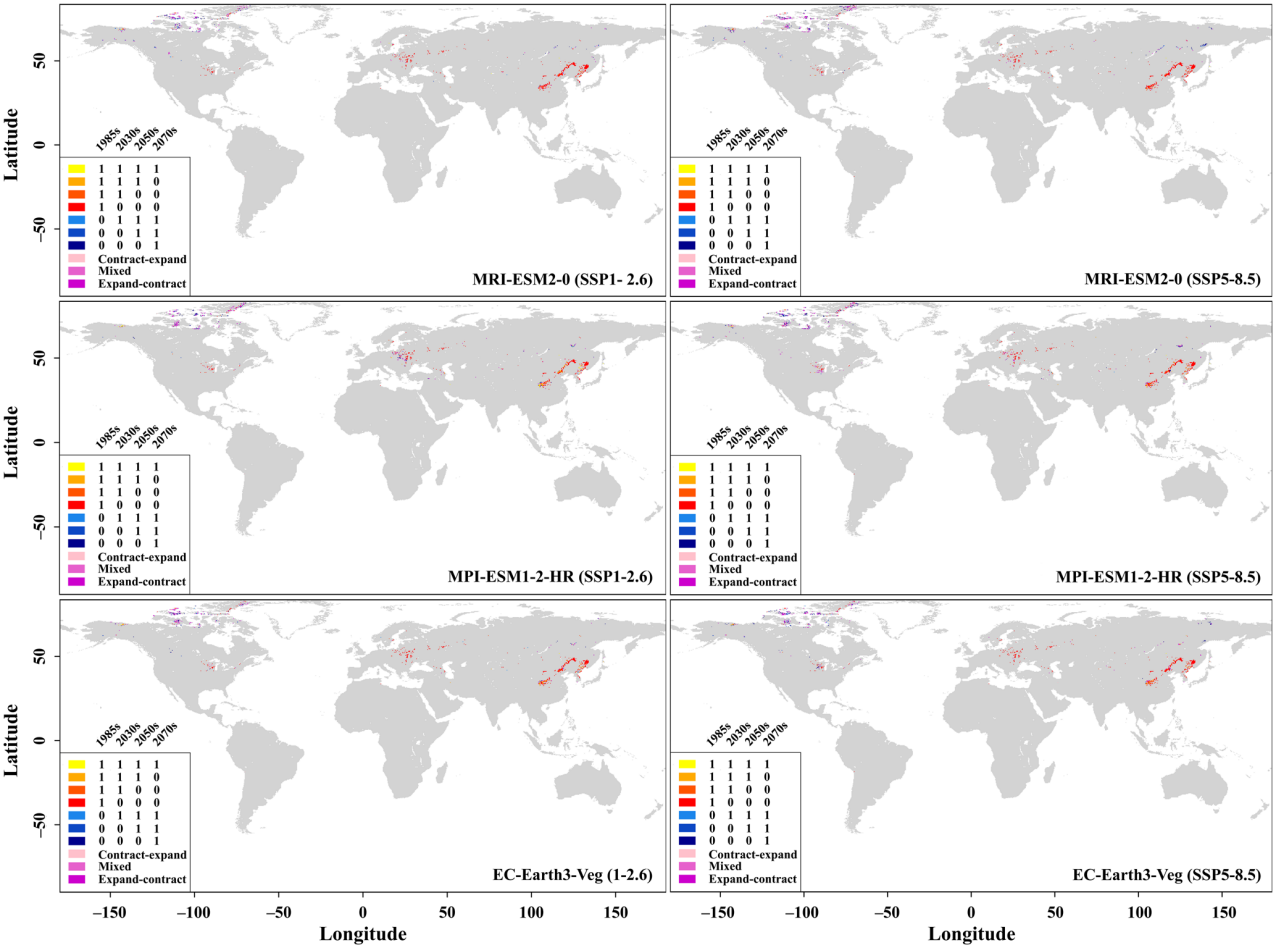
Maps depicting habitat‐suitability's step‐wise expansion (contraction) of *B. lenok tsinlingensis* across multiple global climate models (i.e., MRI‐ESM2‐0, MPI‐ESM1‐2‐HR, and EC‐Earth3‐Veg), scenarios (i.e., SSP1‐2.6 and SSP5‐8.5), and time nodes (i.e., 1985s, 2030s, 2050s, and 2070s). SSP is the abbreviation of shared socio‐economic pathways, while “0” and “1” in the legend indicate unsuitability and suitability, respectively.

According to model prediction, the current PSAs of *B. lenok tsinlingensis* were mainly distributed in China and South Korea of Asia; Ukraine, Poland, Russia, Romania, Sweden of Europe; Alaska, the Mississippi river basin, and northern Canada of North America (Figure 4). However, attributing to growing human activities and global warming, these areas would experience large‐scale shrinkage, and some might even disappear in the future, especially under the background of MRI‐ESM2‐0 and EC‐Earth3‐Veg (Table 2). In terms of this species' future PSAs, the modeling results of all combinations of different GCMs, SSPs, and time nodes were similar: (1) they would expand to some extent in the northern Canada of North America; (2) they would expand sporadically in Alaska and the Mississippi river basin of the United States, Norway, Sweden, Poland, Ukraine, Czechia, Romania, Italy of Europe, as well as Russia, China and South Korea of Asia. Meantime, it is worth noting that in the context of all the three GCMs, the distribution centroids of the fish's PSAs would move northwest (the movement was the least under the combination of MPI‐ESM1‐2‐HR and SSP1‐2.6 (Table 2)). Fortunately, there were always some stable PSAs for the fish in China and South Korea (Figure 4).

**TABLE 2:** Changes of distribution centroids and suitable grid numbers of *B. lenok tsinlingensis* across multiple global climate models, scenarios, and time nodes compared with 1985s (Longitude: 75.6259, Latitude: 46.6609; Suitable grid numbers: 36288).

Global climate models	Scenarios	Time nodes	Distribution centroids		Suitable grid numbers (%)
			Longitude	Latitude	
MRI‐ESM2‐0	SSP1‐2.6	2030s	−9.4961	60.5619	−62.16
		2050s	−33.2992	63.6797	−63.44
		2070s	−41.9922	64.2277	−67.94
	SSP5‐8.5	2030s	−12.7084	60.9246	−60.97
		2050s	−41.5845	65.0671	−68.56
		2070s	−45.0078	68.1658	−63.74
MPI‐ESM1‐2‐HR	SSP1‐2.6	2030s	14.4610	55.4740	−45.83
		2050s	9.4183	56.8290	−53.54
		2070s	8.2270	56.5043	−61.59
	SSP5‐8.5	2030s	32.8815	51.9494	−54.89
		2050s	−23.3624	59.2367	−58.09
		2070s	−41.7744	64.5107	−68.43
EC‐Earth3‐Veg	SSP1‐2.6	2030s	22.2615	54.2312	−55.35
		2050s	1.9763	57.9044	−66.48
		2070s	−17.0755	59.7572	−71.81
	SSP5‐8.5	2030s	11.8273	55.8998	−55.55
		2050s	−15.4111	60.1987	−67.32
		2070s	−52.6123	65.6731	−66.97

*Note:* SSP is the abbreviation of shared socio‐economic pathways.

## 4 Discussion

By species occurrence data and environmental data, we developed a species distribution model for the landlocked piedmont cold‐water fish *B. lenok tsinlingensis*. On the one hand, we hoped to seek out the main environmental driving factors affecting the spatial distribution pattern of this rare species at a global scale. On the other hand, we also expected to unravel how the current PSAs of this endangered species would respond to global climate change and human population density, thereby contributing to the conservation of such fish species. The results showed that: (1) the MaxEnt model had remarkable generalization or transferability ability (AUC > 0.90; Fielding and Bell 1997; Raes and ter Steege 2007) and it is qualified for forecasting the current and future global PSAs of this fish; (2) 7 of the 11 environmental variables used for modeling had significant effects on the spatial distribution of the target species, while only 4 of them to which the fish were sensitive in habitat selection, namely BIO9, Pop, Elev, and BIO8; (3) globally, the PSAs of the lenok would massively shrink and their distribution centroids would shift to the northwest.

### 4.1 Variables' Importance

**FIGURE 5:**
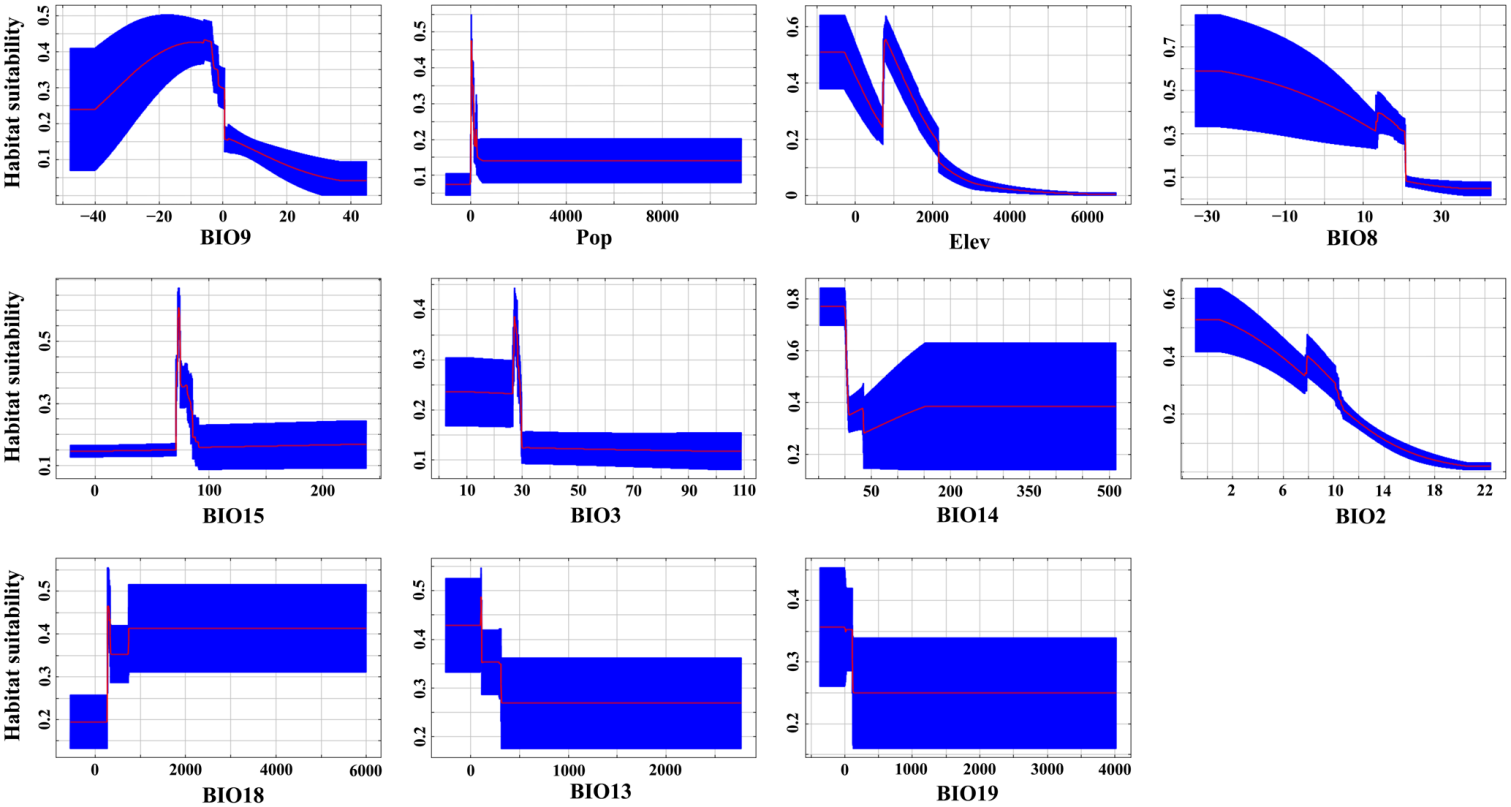
Average response curves (*n* = 10, i.e., 10 repetitions) of 11 variables chosen for MaxEnt modeling. Abbreviations of these environmental variables are described in Table 1. Red lines indicate average values, while blue sustaining slip bands stand for confident intervals of 10 runs.

It is not hard to find that the fish's habitat suitability manifested two distinct response modes to the two important bioclimatic variables used in this study, that is, the marginal response curve to BIO9 exhibited a unimodal distribution as a whole and the maximum appeared at −3°C, whereas the curve to BIO8 showed a decreasing trend. Of course, what is mentionable is that, when BIO8 was between 12°C and 16°C, the habitat suitability showed an increase with a short, sharp, and small extent (Figure 5), which the authors thought might be caused by the close relationship between air and water temperature (Trenberth 2011), since it has been pointed out that the optimum water temperature for the fish was usually between 13°C and 16°C (Hong et al. 2024; Zhang et al. 2022). In other words, when the water temperature exceeds 16°C, with its increase, the habitat suitability will gradually decrease until the habitat is no longer suitable for this species (Hong et al. 2024).

Unlike BIO9 while like BIO8, the response patterns of the fish's habitat suitability to the two abiotic climate variables (i.e., Pop and Elev) in this study were both decreasing overall (Figure 5). Still, slight differences existed between them. For example, the habitat suitability was the most when Pop was 0, then a peak arose near 0 and whereafter continued to decline. As a contrast, there were two peaks in the response curve for Elev (Figure 5). These phenomena are elusive at first glance, but perspicuous if combined with relevant ecological theories and the characteristics of the data used in modeling: (1) according to “the intermediate disturbance hypothesis”, a certain level of disturbance can not only bring about abundant nutrients, but also moderate interspecific competitions, thereby increase population abundance or species diversity, that is, increase habitat suitability (Connell 1978); (2) in this study, we employed the data of *B. lenok tsinlingensis* in both China and South Korea to construct the model, and the former usually live at the altitude of 1500–2000 m (Tao et al. 2024; Wu et al. 2017), while the latter tend to live at the altitude of 234–858 m (Ko et al. 2021), that is, there are two most suitable elevations for this fish.

### 4.2 Current and Future PSAs

According to the available data, *B. lenok tsinlingensis* is now mainly distributed in the alpine streams of the Qinling Mountains of China and the Nakdong River of South Korea (Li 1984; Osinov 2024; Yu and Kwak 2015). Our MaxEnt model predicted that the fish's current PSAs scattered in Asia, Europe, and North America. Nevertheless, under the background of the increasing global warming and human activities, these PSAs would experience large‐scale reduction, and some even might completely vanish in the future (Table 2). As the best out of the worst, the PSAs of the fish in some regions would expand in some degree though compared with in northern Canada, the PSAs' expansion would be quite lesser in the basins of the Alaska and Mississippi Rivers in the United States; Norway, Sweden, Poland, Ukraine, Czechia, Romania, and Italy in Europe; and Russia, China, and South Korea in Asia, especially under MPI‐ESM1‐2‐HR (Figure 4). Meanwhile, the distribution centroids of the PSAs would move northwest. These results are not only similar to those on other cold‐water fish (i.e., their PSAs will be significantly reduced and the distribution centroids will shift to higher altitudes or polar regions; Yu et al. 2013), but also consistent with the field observations on this species by Ren and Liang (2004) and Ko et al. (2011). That is, with the intensifying global warming and human activities, the *B. lenok tsinlingensis*' habitat has been showing a trend of gradually moving to high altitude areas in some regions. It can thus be concluded that, global change will degrade or even devastate *B. lenok tsinlingensis*' habitat and consequently cause adverse impact on biodiversity and wildlife's PSAs (Liu et al. 2022). In summary, *B. lenok tsinlingensis* will probably be in peril in the future.

### 4.3 Management Recommendations

Based on the above and considering its extremely limited dispersion ability (high altitude landlocked piedmont cold‐water fish), special ecological habits (aggregation migration; Deng et al. 2024; Sun 2014), and requirements for high‐quality water (Zhao and Zhang 2009), we put forward management suggestions for this rare and endangered fish as follows: (1) to enhance habitat protection. Natural habitat protection is the key to species conservation, and effective management is necessary for the present habitat of *B. lenok tsinlingensis*. Anthropogenic activities, such as mining, water extraction, damming, and river bank hardening, should be strictly controlled (Bao et al. 2021); (2) to carry out ecological restoration. For those damaged habitats, measures should be taken, such as removing unnecessary dams (Battle et al. 2020), restoring riverbank vegetation, and improving water quality (Tung et al. 2009; Wu et al. 2017) so as to improve the natural reproduction conditions for the fish; (3) to strengthen enforcement of related laws and regulations, such as the Law of the People's Republic of China on the Protection of Wildlife. Illegal fishing, especially fishing with electricity and fishing with poison, should be prohibited; (4) to implement stock enhancement. Designed artificial enhancement and releasing should be carried out to replenish and restore the fish's wild populations. Appropriate times and spots should be selected for releasing, and the health and genetic diversity of released seedlings should be guaranteed (Cheng et al. 2011; Lu 2020); (5) to strengthen scientific research and monitoring. Studies on the ecology, biology, and population dynamics of *B. lenok tsinlingensis* should be fulfilled consistently in order to better understand the species' life history and habitat requirements; (6) to build cross‐regional cooperation. China and South Korea should enhance cooperation to formulate and implement conservation strategies together for the fish; (7) the ultimate and paramount, more global plans are needed to reduce greenhouse gas emissions and increase forest coverage, thereby slowing down global warming. Certainly, based on the modeling results of the present study, the strategy of *ex situ* conservation may be considered to protect the species in the future (i.e., move some individuals to the places where *B. lenok tsinlingensis* can survive).

## 5 Conclusions

Based on its occurrence records and related environmental variables, and by MaxEnt algorithm, the study established a species distribution model for *B. lenok tsinlingensis* at a global scale for the first time. Based on the model, and with the aim to provide a scientific basis for the conservation of this rare and endangered species, we not only deeply explored the driving factors affecting the spatial distribution pattern of this landlocked piedmont cold‐water fish, but also analyzed how its PSAs would respond to global environmental changes. The results showed that: (1) the pivotal environmental factors influencing habitat suitability of this species were BIO9, Pop, Elev, and BIO8; (2) although the model predicted that the current PSAs of this species were distributed in Asia, Europe, and North America, they would reduce on a large scale, and their distribution centroids would shift northwest with the intensification of global changes. Albeit this study came up with some valuable or new insights on how human pressures and global climate change would influence *B. lenok tsinlingensis*, there are still some inadequacies, which should be ameliorated from the following aspects in the future: (1) enriching the explanatory variable set to make the results more comprehensive. For instance, global forest coverage, river connectivity index, and mountain slope aspect data should be added; (2) conducting long‐term real‐time tracking of target species, updating the species occurrence database so as to reduce the heterogeneity of the occurrence records' time and source; (3) optimizing the MaxEnt algorithm, that is, using “kuenm” package in R (Cobos et al. 2019) to debug or analyze the hyperparameters and extrinsic risks such as regularized multipliers (RM) and feature classes (FC); (4) using multiple algorithms (e.g., random forest) or ensemble models for analysis and prediction.

## Author Contributions

**Yuebing Zhou:** conceptualization (equal), data curation (equal), investigation (equal), methodology (equal), software (equal), visualization (equal), writing – review and editing (equal). **Xianghong Dong:** conceptualization (equal), funding acquisition (equal), methodology (equal), project administration (equal), supervision (equal), writing – review and editing (equal). **Tao Ju:** conceptualization (equal), methodology (equal), project administration (equal), supervision (equal), writing – review and editing (equal). **Lei Gan:** data curation (equal), funding acquisition (equal), writing – review and editing (equal). **Zhenlu Wang:** data curation (equal), writing – review and editing (equal). **Yuxi Lian:** data curation (equal), writing – review and editing (equal). **Peng Zhang:** data curation (equal), writing – review and editing (equal). **Xiongfeng Bai:** data curation (equal), writing – review and editing (equal). **Qing Liu:** data curation (equal), writing – review and editing (equal). **Shuhai Zhang:** data curation (equal), writing – review and editing (equal). **Jiyuan Liu:** data curation (equal), writing – review and editing (equal). **Tao Xiang:** data curation (equal), writing – review and editing (equal). **Lei Shi:** data curation (equal), writing – review and editing (equal). **Haibo Jiang:** data curation (equal), writing – review and editing (equal). **Jian Shao:** data curation (equal), funding acquisition (equal), writing – review and editing (equal). **Miao An:** data curation (equal), writing – review and editing (equal).

## Conflicts of Interest

The authors declare no conflicts of interest.

## Data Availability

The original data and source code concerning this research are open access and can be found in Dryad (https://doi.org/10.5061/dryad.f7m0cfz6t).
